# Osteoprotegerin/Receptor Activator of Nuclear Factor-Kappa B Ligand/Receptor Activator of Nuclear Factor-Kappa B Axis in Obesity, Type 2 Diabetes Mellitus, and Nonalcoholic Fatty Liver Disease

**DOI:** 10.1007/s13679-023-00505-4

**Published:** 2023-05-19

**Authors:** Ilias D. Vachliotis, Stergios A. Polyzos

**Affiliations:** 1 First Department of Pharmacology, Medical School, Aristotle University of Thessaloniki, Thessaloniki, 54124 Greece; 2 Second Department of Internal Medicine, 424 General Military Hospital, Thessaloniki, 56429 Greece

**Keywords:** Bone metabolism, Osteoporosis, Nonalcoholic fatty liver disease, Obesity, Type 2 diabetes mellitus

## Abstract

***Purpose of Review*:**

To summarize evidence on the potential involvement of the osteoprotegerin (OPG)/receptor activator of nuclear factor-kappa B (NF-κΒ) ligand (RANKL)/receptor activator of NF-κΒ (RANK) axis in the pathogenesis of metabolic diseases.

***Recent Findings*:**

The OPG-RANKL-RANK axis, which has been originally involved in bone remodeling and osteoporosis, is now recognized as a potential contributor in the pathogenesis of obesity and its associated comorbidities, i.e., type 2 diabetes mellitus and nonalcoholic fatty liver disease. Besides bone, OPG and RANKL are also produced in adipose tissue and may be involved in the inflammatory process associated with obesity. Metabolically healthy obesity has been associated with lower circulating OPG concentrations, possibly representing a counteracting mechanism, while elevated serum OPG levels may reflect an increased risk of metabolic dysfunction or cardiovascular disease. OPG and RANKL have been also proposed as potential regulators of glucose metabolism and are potentially involved in the pathogenesis of type 2 diabetes mellitus. In clinical terms, type 2 diabetes mellitus has been consistently associated with increased serum OPG concentrations. With regard to nonalcoholic fatty liver disease, experimental data suggest a potential contribution of OPG and RANKL in hepatic steatosis, inflammation, and fibrosis; however, most clinical studies showed reduction in serum concentrations of OPG and RANKL.

***Summary*:**

The emerging contribution of the OPG-RANKL-RANK axis to the pathogenesis of obesity and its associated comorbidities warrants further investigation by mechanistic studies and may have potential diagnostic and therapeutic implications.

## Introduction

The prevalence of obesity and its associated comorbidities, mainly type 2 diabetes mellitus (T2DM) and nonalcoholic fatty liver disease (NAFLD), has increased over the last decades [1, 2]. Given their close association with cardiovascular disease (CVD) and all-cause mortality, these metabolic diseases are considered a growing public health burden. Furthermore, except for T2DM, for which a large array of effective and safe pharmacological options have been developed [3], anti-obesity drugs have not meet the desirable expectations [4], while no medications have been approved to-date specifically for the treatment of NAFLD [5]. Better knowledge of the molecular pathways involved in the pathogenesis of these diseases may possibly reveal new molecular targets and may hopefully result in novel therapeutic candidates.

Receptor activator of nuclear factor-kappa B (NF-κΒ) ligand (RANKL), along with its cognate receptor, receptor activator of NF-κΒ (RANK), and osteoprotegerin (OPG), a decoy receptor with high affinity for RANKL, form a molecular system, which has been originally involved in bone remodeling and metabolic bone diseases [6]. Specifically, RANKL, which is produced by osteoblasts, binds to RANK on the surface of osteoclast precursors and promotes osteoclastogenesis and bone resorption. On the other hand, OPG, which is also produced by osteoblasts, attenuates RANKL-RANK interaction through binding to RANKL, thus serving as a negative regulator of osteoclastogenesis and an inhibitor of bone loss. Of note, OPG-expressing osteoblasts have been supported to be a distinct subset of cells from those secreting RANKL, and differentially affect osteoclasts in a paracrine manner [7•]. Disruption of OPG-RANKL-RANK axis in bone has been associated with osteoporosis and other metabolic bone diseases [6].

Recently, a potential role of the OPG-RANKL-RANK axis in metabolic diseases has also emerged. Both obesity and T2DM have been associated with the dysregulation of the OPG-RANKL-RANK axis in bone tissue and subsequently increased risk of low-energy fractures [8••, 9], while a similar association may possibly occur between NAFLD and osteoporosis [10••]. Besides bone, RANKL and OPG are known to be involved also in immune and inflammatory responses, since both are also secreted by T-lymphocytes, and modulate proliferation, activation, and survival of dendritic cells and monocytes/macrophages [11]. In addition to the RANKL-RANK pathway, OPG interferes with the TNF-related apoptosis-inducing ligand (TRAIL)-death receptor (DR) pathway, which is typically involved in inflammation and apoptosis [12]**.** By binding to TRAIL, OPG interrupts TRAIL-DR interaction and thus, it possibly exerts anti-apoptotic effects. Owing to the biological relationship that links OPG and inflammation through regulating the TRAIL and RANKL pathways, emerging evidence suggests a potential role of these systems in metabolic diseases. It is worth noting that metabolic diseases are considered to be, at least partly, a consequence of a systematic low-grade inflammation, which leads to metabolic aberrations, composing the concept of “metabolic inflammation” [13] or “inflammation-induced dysmetabolism.”

This review aims to summarize evidence regarding the potential involvement of the OPG-RANKL-RANK axis in the pathogenesis of metabolic diseases, which may have potential therapeutic implications. In each section, which consecutively refers to obesity, T2DM, and NAFLD, the first part reports data derived from experimental studies, and the second part focuses on evidence from clinical studies.

## Osteoprotegerin/RANKL/RANK in Obesity

### Experimental Studies

Previous studies have supported the existence of complex interactions between the adipose tissue and bones [11]. Obesity is a state of low-grade systematic inflammation, in which pro-inflammatory cytokines, such as tumor necrosis factor-α (TNF-α), interleukin (IL)-1, IL-6, IL-17 [14], and adipokines, such as leptin [15], released from the dysfunctional adipose tissue into the circulation, may regulate the OPG-RANKL-RANK axis in bones, with main final effect, the inhibition of bone formation and the acceleration of bone resorption. Obesity is also associated with increased adipogenesis in bone marrow, which alters the microenvironment of bone tissue. Accumulation of adipocytes in the bone marrow causes a shift from bone formation to bone resorption via stimulation of the OPG-RANKL-RANK system in favor of osteoclastogenesis [16].

Interestingly, expression and production of RANKL and OPG have also been identified in adipocytes [17]; it has been speculated that these molecules may also contribute to the inflammatory process associated with obesity. Indeed, mice on a high-fat diet (HFD) presented increased expression of OPG in the circulation, adipose tissue, pancreas, and the liver [18]. In addition, OPG administration in normal-weighted mice induced inflammatory changes and metabolic disturbances, i.e., increased macrophage recruitment in the adipose tissue, high circulating and adipose tissue levels of pro-inflammatory cytokines, and glucose intolerance [18], which contrasts with the above mentioned potential anti-apoptotic effect of OPG. In line with these observations, OPG -/- mice on HFD demonstrated lower inflammation in the adipose tissue, as reflected by the reduced macrophage infiltration and decreased pro-inflammatory gene expression compared to controls [19•]. Of note, the latter study also provided further mechanistic insights into the role of RANKL in macrophages infiltrating the adipose tissue. More specifically, macrophages present both RANK and toll-like receptor 4 (TLR4) on their surface. These two receptors partly share intracellular signaling molecules, including the adaptor protein TNF receptor-associated factor 6 (TRAF6). In the presence of RANKL, TRAF6 binds to RANK instead of TLR4, even if lipopolysaccharide (LPS) is present [19•]. This indicates that RANKL reduces inflammation in the adipose tissue, at least partly by inhibiting TLR4 activation in macrophages. If elevated OPG exacerbates inflammation by inhibiting RANKL in the adipose tissue [19•], or exerts anti-apoptotic effects through interacting with the TRAIL-DR [12], needs to be shown in further mechanistic studies. RANKL may also be involved in glucose homeostasis, since it increases energy expenditure and improves glucose metabolism by inducing “beiging” of the white adipocytes in the adipose tissue [20]. These emerging data suggest that OPG and RANKL may serve as mediators potentially involved in the pathogenesis of obesity.

### Clinical Studies

Few observational studies have evaluated the association between obesity and the OPG-RANKL-RANK axis with the majority of them focusing on OPG, whereas results for RANKL remain limited; these studies are summarized in Table 1. This may be partly attributed to the technical difficulties that were encountered with the previous kits for RANKL measurement, mainly owing to the fact that serum RANKL constitutes only a small part of total RANKL, as it is mainly cell-bounded and thus not detectable in the circulation [21]. Most studies recruited apparently healthy obese children [22–24], adolescents [24], or young adults [25–27] without other metabolic comorbidities (i.e., metabolically healthy obesity) to show that circulating OPG was lower in obese compared to lean individuals, although some studies showed comparable levels [28, 29] or even increased serum OPG levels in obese than normal-weighted participants [30]. On the other hand, studies which enrolled participants with other metabolic aberrations in the setting of metabolic syndrome (MetS) reported that obese with MetS had higher serum OPG concentrations than controls [18, 31]. Of note, OPG was shown to increase in parallel with the increasing number of metabolic risk factors [31]. Moreover, in a study of 80 elderly overweight or obese adults without diabetes, those with advanced atherosclerosis had higher serum OPG levels than those without it after accounting for potential confounders [32]. Interestingly, there are also some reports, which link specific variants of the OPG [OPG, rs3736228 (AG/AA) variant] and RANK [RANK, rs11664594 (A/T) variant] genes to an increased risk of obesity, which may imply a potential involvement of the OPG-RANKL-RANK axis in the pathogenesis of obesity, but requires validation in independent cohorts of obese individuals [33, 34].

**Table 1:** Summary table of the main clinical studies on the association between the osteoprotegerin/RANKL/RANK axis and obesity^a^

**First author (year) **^**reference**^	**Study design; origin**	**Population characteristics**	**Main findings**
Ugur-Altun et al. (2005) [25]	Case-control; Turkey	*Cases* 50 obese adults without other than obesity metabolic diseases Mean age 31 ± 8 years *Controls* 24 lean adults Mean age 30 ± 7 years	Circulating OPG was lower in obese participants compared with controls. OPG was negatively associated with HOMA-IR.
Ugur-Altun et al. (2007) [26]	Case-control; Turkey	*Cases* 34 obese premenopausal women without other than obesity metabolic diseases Mean age 31 ± 8 years *Controls* 19 lean premenopausal women Mean age 31 ± 7 years	Circulating OPG was lower in obese premenopausal women compared with controls. OPG was negatively associated with HOMA-IR.
Gannage-Yared et al. (2008) [28]	Case-control; Lebanon	*Cases* 102 morbidly obese individuals, candidates for bariatric surgery Mean age 37 ± 11 years *Controls* 64 lean individuals Mean age 36 ± 8 years	Circulating OPG did not differ between obese and non-obese individuals. OPG was positively associated with HOMA-IR and CRP only in obese, but not in non-obese, individuals.
Ashley et al. (2011) [27]	Case-control; Ireland	100 participants without cardiovascular and metabolic diseases: *1st group* 36 lean participants *2nd group* 41 overweight participants *3rd group* 23 obese participants	Circulating OPG was gradually decreased from lean to overweight and then to obese participants. OPG was negatively associated with HOMA-IR and positively with adiponectin. Circulating RANKL did not differ among the 3 groups.
Dimitri et al. (2011) [22]	Case-control; UK	*Cases* 52 obese children Mean age 13 ± 3 years *Controls* 51 lean children Mean age 11 ± 3 years	Circulating OPG was lower in obese children compared with controls. OPG was negatively associated with leptin. Circulating RANKL did not differ between the 2 groups.
Suliburska et al. (2013) [30]	Case-control; Poland	*Cases* 78 obese adolescents Mean age 15 ± 2 years *Controls* 20 lean adolescents Mean age 15 ± 2 years	Circulating OPG was increased in obese adolescents compared with controls. OPG was positively associated with HOMA-IR.
Ayina et al. (2015) [29]	Case-control; Cameroon	*Cases* 44 obese women Mean age 32 ± 5 years *Controls* 16 lean women Mean age 27 ± 6 years	Circulating OPG did not differ between obese and non-obese women. OPG was negatively associated with HOMA-IR and LDL-C and positively with HDL-C in obese women.
Erol et al. (2016) [23]	Case-control; Turkey	*Cases* 107 obese children Mean age 11 ± 3 years *Controls* 37 lean children Mean age 11 ± 3 years	Circulating OPG was lower in obese children compared with controls. No association was found between OPG and HOMA-IR.
Kotanidou et al. (2019) [24]	Case-control; Greece	160 children and adolescents: *Cases* 85 obese individuals: 40 children and 45 adolescents Mean age 12 ± 4 years *Controls* 75 lean individuals: 43 children and 32 adolescents Mean age 11 ± 5 years	Circulating OPG was increased in obese adolescents, but not in obese children, compared with the respective controls. Circulating OPG was increased in obese individuals with IR compared with lean and obese without IR.
Del Toro et al. (2021) [32]	Case-control; Italy	80 obese/overweight participants without diabetes at high CVD risk: *Cases* 55 with advanced atherosclerosis Mean age 70 ± 10 years *Controls* 25 without advanced atherosclerosis Mean age 65 ± 10 years	Circulating OPG was increased in those with compared to those without critical coronary artery and/or carotid artery stenosis, diagnosed by either coronary angiography or US, respectively.

*CRP* C-reactive protein, *CVD* cardiovascular disease, *HDL-C* high-density lipoprotein-cholesterol, *HOMA-IR* homeostasis model assessment - insulin resistance, *IR* insulin resistance, *LDL-C* low-density lipoprotein-cholesterol, *OPG* osteoprotegerin, *RANK* receptor activator of nuclear factor-kappa B, *RANKL* receptor activator of nuclear factor-kappa B ligand, *US* ultrasonography

^a^Studies are sorted according to the year of publication

The above considering, we may speculate that obesity per se may be associated with lower circulating OPG concentrations. However, elevated serum OPG levels in obese may reflect an increased risk of metabolic dysfunction or CVD. An appealing hypothesis may be that, in metabolically healthy obesity, OPG is downregulated as a protective mechanism against the potentially adverse effects of OPG. This counteracting mechanism, however, may be dysregulated when metabolic aberrations are accumulated in obese individuals; thus, OPG is increased and possibly exerts adverse effects. Of course, this remains to be shown by studies of different design. With regard to RANKL, limited existing studies indicated comparable circulating RANKL between obese and lean individuals [22, 27], which, however, needs further verification specifically with high sensitivity kits for measuring serum RANKL.

As mentioned above and suggested by experimental studies, OPG and RANKL are also produced by the adipocytes. However, the exact role of these two molecules in the adipose tissue, their contribution to the pathogenesis of obesity, and their interplay with well-established adipokines, such as leptin and adiponectin, is largely unknown. Increased circulating leptin, which characterizes obesity [35], was correlated with decreased circulating OPG [22]. In bones, leptin directly acts to leptin receptors on the surface of osteoblasts, inhibiting OPG production, which results in increased RANKL concentrations and, subsequently, in increased bone resorption. However, the possible interaction of OPG and leptin in the adipose tissue has not yet been displayed. Furthermore, OPG was shown to be positively correlated with adiponectin [27]. Circulating OPG appears to be decreased in obesity, following a similar pattern to that of adiponectin [36].

## Osteoprotegerin/RANKL/RANK in T2DM

### Experimental Studies

Experimental studies point to an emerging role of the OPG-RANKL-RANK axis not only in the adipose tissue but also in the regulation of glucose homeostasis. Although the molecular mechanisms that link RANKL and OPG with glucose metabolism have not yet been fully elucidated, systemic or hepatic inhibition of RANKL signaling in a mouse model of T2DM ameliorated hepatic insulin resistance (IR), one of the main pathogenic key factors of T2DM, and markedly improved serum glucose concentrations [37]. Moreover, a recent study suggested a potential role of the OPG-RANKL-RANK axis in muscle metabolism, as RANKL promoted IR in muscle cells, while RANKL inhibition, either with denosumab (Dmab) or with OPG immunoglobulin fragment complex (OPG-Fc), resulted in the improvement of muscle strength, insulin sensitivity, and glucose uptake [38]. Of note, Dmab, a human monoclonal IgG2 antibody, which mimics the biological functions of OPG, by blocking RANKL, but not TRAIL, is an established medication for osteoporosis and other metabolic bone diseases [39].

Another potential mechanism by which the OPG-RANKL-RANK system may regulate glucose and insulin metabolism was proposed by a recent study, in which recombinant OPG administration in diabetic mice significantly improved glucose homeostasis by increasing β-cell mass [40]. Notably, in vitro and in vivo studies have identified the expression of OPG, RANKL, and RANK also in the pancreatic human β-cells [41, 42]. The RANKL-RANK pathway was demonstrated to function as an inhibitor of β-cell proliferation in both mice and human islets, an effect that was reversed by OPG, which stimulates β-cell proliferation by inhibiting RANKL-RANK interaction, thus acting as a β-cell mitogen [40]. Additionally, TNF-α, IL-1, and LPS have been shown to induce OPG production by pancreatic β-cells, which, in turn, restricts insulin secretion and improves their survival [42]; this may mean that OPG targets to protect the survival of β-cells with the cost of hypoinsulinemia under inflammatory circumstances, an hypothesis that may warrant further research. Nevertheless, the beneficial effects of OPG on pancreatic β-cells and glucose metabolism were not shown by other studies [43, 44], so this issue warrants further investigation.

**Fig. 1:**
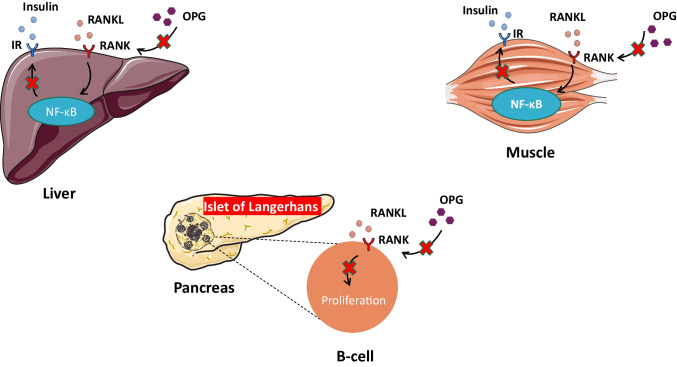
The proposed role of the OPG-RANKL-RANK axis in main organs contributing to glucose metabolism. In the pancreas, the RANKL-RANK signaling pathway inhibits β-cell proliferation, while OPG, by blocking this interaction, stimulates pancreatic β-cell proliferation. In the liver, the RANKL-RANK pathway potentiates hepatic insulin resistance through activating the NF-κB. In contrast, inhibition of hepatic RANKL by OPG or anti-RANKL treatment may ameliorate hepatic insulin resistance and improve serum glucose levels. Similarly, in muscle cells, RANKL promotes insulin resistance, while RANKL inhibition by OPG or anti-RANKL treatment may result in improvement of insulin sensitivity, glucose uptake, and muscle strength. Abbreviations: IR, insulin resistance; NF-κB, nuclear factor-kappa B; OPG, osteoprotegerin; RANK, receptor activator of nuclear factor-kappa B; RANKL, receptor activator of nuclear factor-kappa B ligand

Collectively, these findings propose RANKL and OPG as potential regulators of glucose metabolism by acting either in the pancreas or in peripheral tissues. Figure 1 illustrates the potential role of the OPG-RANKL-RANK axis in the regulation of glucose metabolism. In particular, OPG may act locally in the pancreas probably as a protective factor for β-cells, prolonging survival and preventing the exhaustion of their endocrine function, especially under inflammatory conditions, while RANKL signaling appears to have a potential adverse effect on β-cells function. Additionally, RANKL signaling seems to impair insulin sensitivity in peripheral tissues, including the liver and skeletal muscles. Therefore, dysregulation of the OPG-RANKL-RANK system may represent a potential contributor to the pathogenesis of T2DM.

### Clinical Studies

Clinical studies on OPG-RANKL-RANK axis in T2DM patients are summarized in Table 2. In clinical terms, T2DM has been consistently associated with increased serum OPG concentrations [45], while some anti-diabetic medications, i.e., rosiglitazone, but not metformin, have been shown to reduce them [46]. Circulating OPG levels increased gradually from healthy controls to patients with pre-diabetes [47, 48] or early onset T2DM [49, 50] and even more in diabetic patients with longer disease duration [51, 52]. Among patients with established T2DM, circulating OPG was elevated in those with poor glycemic control compared to those with adequate glucose control [53]. Interestingly, increased circulating OPG has been proposed as a potentially useful biomarker for predicting loss of glycemic control among patients with T2DM [54].

**Table 2:** Summary table of the main clinical studies on the association between the osteoprotegerin/RANKL/RANK axis and T2DM^a,b^

**First author (year) **^**reference**^	**Study design; origin**	**Population characteristics**	**Main findings**
**Association between OPG and T2DM**			
Anand et al. (2006) [56]	Prospective cohort; UK	510 T2DM patients without overt CVD Mean age 53 ± 8 years Diabetes duration 8 ± 6 years Follow-up 18 ± 5 months	Circulating OPG was positively associated with coronary artery calcification score, a marker of subclinical CAD, and could predict short-term cardiovascular events.
Ishiyama et al. (2009) [58]	Case-control; Japan	*Cases* 168 T2DM patients Mean age 62 ± 9 years Diabetes duration 10 ± 8 years *Controls* 40 controls Mean age 60 ± 6 years	No significant difference in circulating ΟPG between T2DM patients and controls. Circulating OPG was positively associated with IMT, a marker of subclinical atherosclerosis, in T2DM.
Xiang et al. (2009) [66]	Cross-sectional; China	154 newly diagnosed T2DM patients: 88 with normoalbuminuria, 41 with microalbuminuria and 25 with macroalbuminuria Mean age 62 ± 11 years Diabetes duration NA	Circulating OPG was gradually increased from normoalbuminuric to microalbuminuric and then to macroalbuminuric group. OPG was positively associated with urinary albumin excretion and negatively with flow-mediated dilation, a marker of endothelial function.
Nabipour et al. (2010) [52]	Case-control; Iran	382 postmenopausal women: *Cases* 102 T2DM patients Mean age 60 ± 7 years Diabetes duration NA *Controls* 280 controls Mean age 58 ± 8 years	Circulating OPG was increased in T2DM postmenopausal women compared with controls.
Nybo et al. (2010) [69]	Cross-sectional; Denmark	305 T2DM patients: 57 with and 248 without peripheral neuropathy, respectively Mean age 65 ± 11 and 57 ± 11 years, respectively Diabetes duration 32.2 ± 5 and 32.3 ± 6 years, respectively	Circulating OPG was increased in T2DM patients with peripheral neuropathy compared with those without.
Reinhard et al. (2010) [64]	Prospective cohort; Denmark	283 T2DM patients Mean age 54 ± 9 years Diabetes duration NA Follow-up: 17 (0.2–23) years	Increased circulating OPG could predict all-cause mortality, independently from other conventional cardiovascular risk factors.
Poulsen et al. (2011) [59]	Cross-sectional; Denmark	305 T2DM patients without known or suspected CVD Mean age 59 ± 11 years Diabetes duration 4.5 ± 5.3 years	Increased circulating OPG was associated with subclinical carotid artery disease and PAD, but not with CAD.
Reinhard et al. (2011) [57]	Cross-sectional; Denmark	200 T2DM patients with microalbuminuria without history of CVD Mean age 59 ± 9 years Diabetes duration 13 ± 7 years	Increased circulating OPG was associated with asymptomatic significant CAD, defined by abnormal MPI and/or stenosis on coronary angiography.
Altinova et al. (2011) [53]	Cross-sectional; Turkey	166 T2DM patients Mean age 57 ± 1 years Diabetes duration 9 (4–13) years	Circulating OPG was increased in poorly-controlled T2DM patients (HbA1c ≥ 7%) compared with well-controlled T2DM patients (HbA1c < 7%). OPG was positively correlated with serum glucose levels, HbA1c, HOMA-IR and microalbuminuria.
Chang et al. (2011) [65]	Cross-sectional; Taiwan	179 T2DM patients: 68 with normoalbuminuria, 67 with microalbuminuria, and 44 with macroalbuminuria Mean age 61 ± 11, 64 ± 11, and 62 ± 11, respectively Diabetes duration NA	Circulating OPG was gradually increased from normoalbuminuric to microalbuminuric and then to macroalbuminuric group.
Aoki et al. (2013) [76]	Cross-sectional; Japan	124 T2DM patients without advanced diabetic nephropathy Mean age 66 ± 8 years Diabetes duration 14.7 ± 8.2 years	Increased circulating OPG was associated with increased cervical artery calcification, measured by US.
Tavintharan et al. (2014) [74]	Cross-sectional; Singapore	1220 T2DM patients Mean age 57 ± 11 years Diabetes duration 11.2 ± 8.9 years	Increased circulating OPG was associated with microvascular complications (nephropathy, neuropathy, retinopathy), but not PAD.
Niu et al. (2015) [50]	Cross-sectional; China	599 with NGR, 730 with IGR and 327 newly-diagnosed T2DM patients Mean age 54 ± 8, NA for the whole subgroup, and 57 ± 8 years, respectively Diabetes duration NA	Patients with T2DM or IGR had increased circulating OPG compared with individuals with NGR. Circulating OPG increased gradually from participants with normal albumin excretion to those with microalbuminuria and then to those with macroalbuminuria.
Niu et al. (2015) [63]	Cross-sectional; China	712 T2DM patients: 505 with and 207 without lower extremity arterial disease Mean age 65 ± 11 and 54 ± 12 years, respectively Diabetes duration NA	Increased circulating OPG was independently associated with the presence and severity of lower extremity arterial stenosis, diagnosed by US.
Yu et al. (2015) [71]	Case-control; China	*Cases* 254 T2DM patients (100 without diabetic retinopathy, 90 with proliferative diabetic retinopathy, and 64 with non-proliferative diabetic retinopathy) Mean age 57 ± 12, 55 ± 12, and 55 ± 12, respectively Diabetes duration NA *Controls* 62 controls Mean age 57 ± 9 years	Increased serum and vitreous OPG were associated with the presence and severity of diabetic retinopathy.
Moh et al. (2020) [54]	Prospective cohort; Singapore	674 T2DM patients with controlled diabetes (HbA1c < 8%) at baseline in the prospective analysis Mean age 59 ± 10 years Diabetes duration NA Follow-up 3 years	Baseline OPG predicted worsening of glycemic control and progression of albuminuria.
**Association between RANKL/ RANK and T2DM**			
Bourron et al. (2014) [80]	Cross-sectional; France	198 T2DM patients at high CVD risk, without severe kidney disease Mean age 64 ± 8 years Diabetes duration 15 ± 10 years	Neither circulating RANKL nor OPG were associated with lower limb arterial calcification score, measured by CT.
Bourron et al. (2020) [81]	Prospective cohort; France	163 T2DM patients at high CVD risk, without severe kidney disease Median age 65 (58–70) years Diabetes duration 12 (6–20) years Follow-up 31 ± 4 months	Circulating RANKL and RANKL/OPG, but not OPG, were associated with the progression of lower limb arterial calcification, measured by CT.
Nita et al. (2021) [73]	Cross-sectional; Romania	171 T2DM with a relatively good glycemic control Mean age 61 ± 10 years Diabetes duration 7.7 ± 6.7 years	Circulating RANKL was lower in those with than without macrovascular complications. Circulating RANKL was not associated with peripheral neuropathy.

*CAD* coronary artery disease, *CVD* cardiovascular disease, *CT* computed tomography, *HbA1c* glycated hemoglobin, *HOMA-IR* homeostasis model assessment - insulin resistance, *IMT* intima-media thickness, *IGR* impaired glucose regulation, *MPI* myocardial perfusion imaging, *NA* not available, *NGR* normal glucose regulation, *OPG* osteoprotegerin, *PAD* peripheral artery disease, *RANK* receptor activator of nuclear factor-kappa B, *RANKL* receptor activator of nuclear factor-kappa B ligand, *T2DM* type 2 diabetes mellitus, *US* ultrasonography

^a^Only studies with sample size > 100 participants were included

^b^Studies are sorted according to the year of publication

Furthermore, increased circulating OPG was associated with diabetic complications and increased in parallel with their severity [51]. More specifically, several studies have shown association of increased circulating OPG with worsened macrovascular complications in T2DM, including coronary artery disease [55–58], carotid artery disease [58–61], and peripheral artery disease [59, 62, 63]. Of note, one study reported that increased circulating OPG could predict all-cause mortality in patients with T2DM [64]. In addition, microvascular complications of T2DM, such as diabetic nephropathy [65–67], diabetic neuropathy [68, 69], and diabetic retinopathy [70, 71], have also been associated with increased plasma OPG concentrations.

The source of the observed increase in circulating OPG in T2DM remains largely unknown. In fact, OPG may be derived from several sources, e.g., bone, pancreas, and blood vessels. Hyperglycemia has been speculated to increase circulating OPG [72] and reduce RANKL concentrations [73]. Moreover, IR has been also associated with increased serum OPG levels [74]. Intriguingly, studies including non-diabetic individuals suggest that circulating OPG is negatively associated with IR, possibly implying that insulin may reduce serum OPG concentrations, or that OPG may reduce insulin concentrations. However, this association seems to be reversed, since their association becomes positive in certain conditions with advanced IR, such as T2DM [27], which, however, requires studies of different design to be validated. It is also important to underline that increased circulating OPG may originate from the atherosclerotic vessels, given the consistent positive association of OPG with vascular calcification (VC), a process that is accelerated in diabetic patients [75]. OPG is produced in large quantities by endothelial and vascular smooth muscle cells, and possibly acts locally, since its tissue concentrations are 500 times greater than plasma concentrations [27]. It should be also highlighted that RANK and RANKL expression are also observed in atherosclerotic lesions, but not in healthy vessels [76]. Actually, elevated circulating OPG and RANKL may reflect an active calcifying process, which is propagated in the setting of T2DM, raising the possibility of the existence of a bone-vascular axis. However, contrary to the known functions of OPG and RANKL at bone metabolism, RANKL seems to increase calcification in the vasculature, whereas OPG blocks this effect [77, 78], indicating a differential effect of the OPG-RANKL-RANK signaling in vascular compared to bone metabolism.

Findings on the association between RANKL and T2DM are less conclusive since some studies have reported decreased circulating RANKL in diabetic patients in comparison to non-diabetic individuals [49, 61], whereas other authors failed to demonstrate any difference [79]. In addition, although an observational cross-sectional study did not show an association between circulating RANKL and peripheral artery disease in T2DM patients [80], the same authors demonstrated that circulating RANKL, but not circulating OPG, was associated with the progression of lower limb arterial calcification in a prospective observational study [81]. Intriguingly, it has also been suggested that increased circulating RANKL may precede T2DM onset and possibly serve as a predictor of T2DM development, an hypothesis needing to be validated, and that OPG concentrations may not precede T2DM, but rather emerge as T2DM occurs, potentially as a compensatory mechanism, which is consistent with the findings from experimental studies [37]. Beyond this hypothesis, circulating OPG may only reflect diabetic vascular complications, and thus are increasingly higher as diabetes worsens overtime. Further studies are needed to clarify the role of the OPG-RANKL-RANK axis in T2DM.

## Osteoprotegerin/RANKL/RANK in NAFLD

### Experimental Studies

Another emerging topic is the potential implication of the OPG-RANKL-RANK axis in the pathogenesis of NAFLD [82]. Mice on HFD, which represents an experimental model of obesity and NAFLD, were initially shown to have not only increased circulating OPG but also increased OPG gene expression in the liver [18]. However, other authors showed that mice on HFD have lower circulating ORG and higher RANKL than control mice [83]. In line, another group reported that HFD caused a gradual increase in circulating RANKL levels and hepatic RANKL expression from controls to mice with simple nonalcoholic fatty liver (NAFL) and then to mice with nonalcoholic steatohepatitis (NASH), regarded as a more severe than NAFL phenotype of the disease [84]. This study, importantly, provided some interesting mechanistic insights, as it showed in vitro that the expression of runt-related transcription factor 2 (Runx2) regulated the production of RANKL in hepatic stellate cells (HSCs), which could subsequently mediate macrophage infiltration in the liver [84]. Intriguingly, a transgenic mouse model of osteoporosis (TgHuRANKL), which overexpresses human RANKL, may develop NAFLD [85].

These experimental data suggest that hepatic expression of RANKL may potentially be upregulated in NAFLD, while relevant data on hepatic OPG are still contradictory; however, the exact source, role, and regulation of these molecules in the liver are largely unknown. HSCs and more specifically their activated type (myofibroblasts) have been proposed as the main source of OPG in the liver, linking OPG to hepatic fibrogenesis [86]. Notably, transforming growth factor-β (TGF-β) and IL-13, two important mediators of hepatic fibrogenesis were shown to induce OPG production in murine liver tissue [87] and, vice versa, OPG was shown to enhance fibrosis by stimulating TGF-β production in the liver, thus creating a local vicious loop, possibly contributing to hepatic fibrinogenesis [86]. In addition, OPG seems to affect hepatic steatosis, as its overexpression triggers the signal-regulated kinase (ERK)-peroxisome proliferator-activated receptor-γ (PPAR-γ)-cluster of differentiation (CD36) pathway and, therefore, resulted in increased hepatic lipid accumulation, highlighting the potential pleiotropic effects of OPG in liver disease [88].

Collectively, limited data support that hepatic OPG may favor hepatic steatosis, NASH, and fibrosis, while hepatic RANKL upregulation may be related to persistent hepatic inflammation and hepatocellular injury. However, more data are needed to consolidate or not these findings.

### Clinical Studies

Clinical studies on the OPG-RANKL-RANK axis in NAFLD patients are summarized in Table 3. Contrary to the above mentioned experimental studies, NASH, but not NAFL, was associated with lower circulating OPG compared to non-NAFLD participants [89], whereas two subsequent case-control studies with biopsy-proven NAFLD supported a gradual decrease of serum OPG levels from controls to patients with NAFL and then to NASH patients [90, 91]. In another case-control study of patients with T2DM, those with concomitant ultrasound-defined NAFLD had lower circulating OPG than those without [92]. Similarly, OPG was shown to be lower in obese children with NAFLD compared to non-NAFLD obese children [93]. Additionally, a more recent study showed reduced circulating OPG together with reduced mRNA and serum levels of RANKL in NAFLD patients compared to healthy controls [94]. Consistent with this, gene expression and plasma concentration of RANK were also reported to be downregulated in NAFLD patients in comparison to healthy subjects [95]. However, few studies have also supported either comparable circulating OPG levels between patients with and without NAFLD [23, 96] or increased serum OPG levels in NAFLD patients when compared to non-NAFLD individuals [97]. Of note, one study including participants with at least one MetS criterion, but not exclusively NAFLD patients, supported the existence of a positive association between circulating OPG and hepatic fat content, which is in line with experimental studies linking OPG with hepatic steatosis [98].

**Table 3:** Summary table of the main clinical studies on the association between the osteoprotegerin/RANKL/RANK axis and NAFLD^a^

**First author (year) **^**reference**^	**Study design; origin**	**Population characteristics**	**Main findings**
**Association between OPG and NAFLD**			
Yilmaz et al. (2010) [89]	Case-control; Turkey	*Cases* 99 biopsy-proven NAFLD patients: 17 NAFL, 26 borderline NASH and 56 definite NASH Mean age 48 ± 10, 49 ± 11, 45 ± 12 years, respectively *Controls* 58 controls without NAFLD Mean age 47 ± 9 years	Circulating OPG was lower in patients with NASH, but not NAFL, compared with controls. OPG was negatively associated with HOMA-IR and aminotransferases.
Ayaz et al. (2014) [97]	Case-control; Turkey	*Cases* 60 US-defined NAFLD patients 70% steatosis grade I, 30% steatosis grade II Median age 45 (24–65) years *Controls* 30 controls without NAFLD Median age 40 (24–57) years	Circulating OPG was increased in patients with NAFLD compared with controls. OPG was positively associated with CIMT in patients with NAFLD. OPG could predict CIMT increase after adjustment for potential confounders.
Yang et al. (2015) [90]	Case-control; China	*Cases* 179 biopsy-proven NAFLD patients: 52 NAFL, 59 borderline NASH, and 68 definite NASH Mean age 30 ± 12 years *Controls* 91 without liver disease, 45 ALD, 50 HBV, 52 HCV Mean age 29 ± 7, 38 ± 8, 29 ± 7, 30 ± 16, 35 ± 19 years, respectively	Circulating OPG was gradually decreased from controls to patients with NAFL and then to patients with NASH. Circulating OPG levels did not differ between patients with ALD, HBV, HCV, and controls.
Niu et al. (2016) [92]	Case-control; China	*Cases* 367 T2DM patients with US-defined NAFLD Mean age 59 ± 13 years *Controls* 379 T2DM patients without NAFLD Mean age 64 ± 12 years	Circulating OPG was lower in T2DM patients with NAFLD compared with controls. OPG was negatively associated with aminotransferases.
Oguz et al. (2016) [96]	Case-control; Turkey	*Cases* 41 US-defined NAFLD patients at low-risk of CVD Mean age 38 ± 9 years *Controls* 37 controls without NAFLD Mean age 35 ± 9 years	Circulating OPG did not differ between patients with NAFLD and controls. OPG was not associated with markers of subclinical atherosclerosis.
Erol et al. (2016) [23]	Case-control; Turkey	107 obese children Mean age 11 ± 3 years *Cases* 62 obese children with US-defined NAFLD *Controls* 45 obese children without NAFLD	Circulating OPG did not differ between obese children with and without NAFLD.
Yang et al. (2016) [91]	Case-control; China	*Cases* 136 biopsy-proven NAFLD patients Mean age NA *Controls* 83 controls with US-defined non-NAFLD Mean age NA	Circulating OPG was positively associated with hepatocyte ballooning, intralobular inflammation and fibrosis stage, and negatively with NAFLD activity score (NAS). Circulating OPG was lower in NASH patients compared with NAFL patients.
Amrousy et al. (2020) [93]	Case-control; Egypt	*Cases* 40 obese children with US-defined NAFLD Mean age 12 ± 1 years *Controls* 40 obese children without NAFLD Mean age 12 ± 1 years	Circulating OPG was lower in obese children with NAFLD compared with non-NAFLD obese children. OPG was negatively associated with ALT and TNF-α.
**Association between RANKL/RANK and NAFLD**			
Mantovani et al. (2019) [100]	Cross-sectional; Italy	77 postmenopausal women with T2DM Mean age 72 ± 8 years *1st group* 15 controls *2nd group* 52 patients with US-defined NAFLD *3rd group* 10 patients with NAFLD and significant hepatic fibrosis measured by Fibroscan	Circulating RANKL was gradually decreased from controls to patients with NAFLD but without significant fibrosis, and then to patients with NAFLD and significant fibrosis.
Niksersht et al. (2020) [94]	Case-control; Iran	*Cases* 57 men with US-defined NAFLD Mean age 44 ± 9 years *Controls* 25 men without NAFLD Mean age 37 ± 9 years	Circulating OPG and RANKL were lower in NAFLD men compared with controls. OPG and RANKL expression were lower in NAFLD men compared with controls.
Hadinia et al. (2020) [95]	Case-control; Iran	*Cases* 63 patients with NAFLD Mean age NA *Controls* 25 apparently healthy Mean age NA	Circulating and mRNA levels of RANK were lower in NAFLD patients compared with controls.

*ALD* alcoholic liver disease, *ALT* alanine aminotransferase, *CIMT* carotid intema-media thickness, *CVD* cardiovascular disease, *HBV* hepatitis B virus, *HCV* hepatitis C virus, *HOMA-IR* homeostasis model assessment - insulin resistance, *NA* not available, *NAFLD* nonalcoholic fatty liver disease, *NAFL* nonalcoholic fatty liver, *NASH* nonalcoholic steatohepatitis, *OPG* osteoprotegerin, *RANK* receptor activator of nuclear factor-kappa B, *RANKL* receptor activator of nuclear factor-kappa B ligand, *TNF-α* tumor necrosis factor-α, *T2DM* type 2 diabetes mellitus, *US* ultrasonography

^a^Studies are sorted according to the year of publication

Taken together, most studies showed reduction in serum concentrations of OPG and RANKL in patients with NAFLD, whose pathophysiological explanation, if any, remains obscure. Moreover, to-date, any effort to explain the discrepancy in OPG and RANKL between experimental and clinical studies in NAFLD is considered to be insecure. It seems that OPG follows the pattern observed in T2DM in animal NAFLD, i.e., it increases with the disease severity, whereas OPG follows the pattern observed in obesity in human NAFLD, i.e., it decreases with disease severity. As mentioned above, apart from being a decoy receptor for RANKL, OPG also operates as a trap receptor for TRAIL, a major apoptotic factor for hepatocytes. Consequently, OPG depletion potentiates apoptosis, which is a hallmark of NASH. However, this speculation remains to be shown specifically for NASH. In agreement with this scenario, high serum OPG and low serum RANKL levels have been reported in advanced fibrosis or cirrhosis of various etiologies [99•], including also NAFLD-related fibrosis [100]. In this regard, OPG has been proposed as a promising biomarker of liver fibrosis [99•], which also remains to be validated. Hepatic RANKL may probably follow an opposite direction than OPG, but it also remains to be definitely established in NAFLD.

## Conclusion

OPG and RANKL, traditionally included in osteokines and playing an important role in bone metabolism, are now increasingly recognized to be involved in the pathogenesis of chronic metabolic diseases, based on emerging experimental evidence. In the clinical setting, most observational studies showed low OPG concentrations in metabolically healthy obesity and NAFLD, whereas high concentrations in T2DM, in which higher OPG was also associated with the severity of disease and diabetic complications. In addition, RANKL seems to adversely affect glucose metabolism and may be positively associated with NAFL and NASH.

This topic has certain challenges, perspectives, and clinical implications. Firstly, determination of circulating OPG and RANKL remains challenging, since they may originate from different tissues and most importantly, their circulating concentrations may largely differ from those on distinct tissue levels (e.g., bone, adipose tissue, liver, vessels). A recent study suggested that OPG and RANKL functions are restricted exclusively at their production sites [7•], highlighting the importance of tight local control of the OPG-RANKL-RANK network in various tissues, while measurements of circulating OPG or RANKL may be not clinically relevant, since they may possibly be an epiphenomenon, which, however, needs to be verified by subsequent studies. Furthermore, commercially available kits for circulating RANKL or OPG do not always provide optimal results, especially older ELISA kits for RANKL, which creates the need for newer kits for more accurate measurement of circulating OPG and RANKL concentrations.

In therapeutic terms, Dmab, an anti-RANKL medication approved for the treatment of osteoporosis, may also prove suitable in the future for the treatment of metabolic diseases, e.g., obesity, T2DM, and NAFLD. One-year administration of Dmab in T2DM patients with osteoporosis improved glycated hemoglobin (HbA1c), homeostasis model assessment – IR (HOMA-IR), an index of IR, and liver function tests [101]. Another prospective study demonstrated that a single dose of Dmab was effective in reducing HbA1c and hepatic insulin resistance index in postmenopausal women with osteoporosis [102]. Similarly, a recent meta-analysis reported that Dmab improved glycemic parameters, mainly in patients with impaired glucose tolerance, such as those with pre-diabetes or diabetes [103••]. Moreover, administration of Dmab to a woman with osteoporosis and concomitant NASH improved her liver function tests, which deserves further investigation [104]. We previously hypothesized Dmab repurposing in NAFLD [105•], and, to this aim, we are currently running a non-sponsored clinical study with Dmab administration in patients with osteoporosis and NAFLD (clinicaltrials.gov identifier: 88235).

In conclusion, this review summarizes current evidence on the potential contribution of the OPG-RANKL-RANK axis to the pathogenesis of metabolic diseases (obesity, T2DM, and NAFLD). Although the topic seems to be challenging, further mechanistic studies are needed to shed light in the definite implication of OPG and RANKL in the pathophysiology of metabolic diseases. Diagnostic accuracy studies are also warranted to show whether OPG and RANKL may serve as predictors of metabolic diseases or their severity, as well as clinical trials to show the efficacy of anti-RANKL treatment in metabolic diseases beyond the bone.

## Data Availability

No datasets were generated or analyzed during the current review article.
